# Comparative evaluation of three dengue duo rapid test kits to detect NS1, IgM, and IgG associated with acute dengue in children in Myanmar

**DOI:** 10.1371/journal.pone.0213451

**Published:** 2019-03-13

**Authors:** Woong Sik Jang, Seung Yeon Kwak, Win Lai May, Dong June Yang, Jeonghun Nam, Chae Seung Lim

**Affiliations:** 1 Department of Emergency Medicine, College of Medicine, Korea University Guro Hospital, Seoul, Republic of Korea; 2 Department of Laboratory Medicine, College of Medicine, Korea University Guro Hospital, Seoul, Republic of Korea; 3 Department of Medical Research, Ministry of Health and Sports, Dagon Township, Yangon, Myanmar; Academia Sinica, TAIWAN

## Abstract

Dengue is an increasing public health concern worldwide and requires efficient laboratory diagnostics. We evaluated three commercially available dengue rapid diagnostic tests—the Humasis Dengue Combo NS1 & IgG/IgM (Humasis, Korea), SD Bioline Dengue Duo NS1 Ag & IgG/IgM (SD Bioline, Korea), and CareUS Dengue Combo NS1 and IgM/IgG kits (WellsBio, Korea)—and compared them to reference immunoglobulin M (IgM) or immunoglobulin G (IgG) ELISAs and quantitative reverse transcription polymerase chain reaction (qRT-PCR) assays. In total, 109 dengue-positive samples from children with acute symptomatic dengue and 63 dengue-negative samples from febrile and asymptomatic individuals were collected. For the nonstructural 1 protein (NS1) Ag test, the sensitivity and specificity were in the following order: CareUS (79.82 and 100%), Humasis (63.30 and 100%), and SD Bioline (48.62 and 100%). For IgM and IgG, CareUS had the highest sensitivities and specificities (89.91 and 100%; 82.57 and 100%, respectively), followed by SD Bioline (60.55 and 100%, 77.98 and 100%, respectively), and Humasis (51.38 and 98.21%, 72.48 and 95.24%, respectively). The IgM kits were more sensitive than the NS1 Ag or IgG kits; however, combining NS1 Ag and IgM reduced the number of missed cases. Therefore, the NS1 Ag plus IgM dengue kits increase the accuracy of the results. In our study, the CareUS Dengue Combo NS1 and IgM/IgG kit showed higher accuracy in performance with reference to qRT-PCR and ELISA results.

## 1. Introduction

Dengue fever is caused by four serotypes of dengue virus (DENV)—DENV1, DENV2, DENV3, and DENV4—and is transmitted mainly by *Aedes aegypti*. Since the end of the 20th century, dengue infection has been found to be common in subtropical areas, and has also been reported in urban areas [1, 2]. Prior to 1970, severe forms of DENV infection, such as dengue hemorrhagic fever (DHF) and dengue shock syndrome (DSS), were restricted to only nine countries [3]. However, the disease is now endemic in more than 100 countries, including countries in Africa, the Americas, the Mediterranean region of the Middle East, Southeast Asia, and the Western Pacific [3, 4]. In 2015, 2.35 million cases of dengue were reported in the Americas alone, of which 10,200 cases were diagnosed as severe dengue and led to 1,181 deaths [5]. The World Health Organization (WHO) estimates 390 million people are infected with dengue fever annually [6], resulting in 500,000 hemorrhagic dengue fevers that leads to 25,000 deaths every year [7]. In particular, it has been reported that dengue infection in Myanmar predominately occurs in children aged less than 15 (97% and 98% respectively) with a total of 89,832 cases and 393 hospital deaths occurring between 2011 and 2015 [8]. Tragically, DHF is a leading cause of hospitalization and death of children in several Asian countries. Case-fatality rates, although normally 2.5%, can exceed 20%, but can be reduced to < 1% with rapid recognition and proper treatment. Therefore, the early management of patients with dengue infections is essential to prevent the occurrence of severe forms of the disease, and the ability to rapidly confirm an acute dengue infection could aid in providing the accurate treatment and management of patients as early as possible [9].

The early diagnosis of patients with dengue is critical for timely clinical intervention, etiological investigations, and in disease control [10]. The WHO 2009 guidelines suggest the use of a variety of methods for dengue diagnosis, including virus isolation, nucleic acid detection, detection of antigens, serological tests, hematological tests, viral isolation and identification, nucleotide detection, and serological tests for IgM or IgG seroconversion. However, these methods have limitations in terms of their ability to be used for immediate diagnosis in the field, since they require expertise, sophisticated facilities, expensive laboratory equipment, a considerable amount of time for viral isolation and RNA purification, and the availability of additional serum samples to confirm serological tests.

Recently, based on immunochromatographic or immunoblot technologies, commercially available DENV kits have been developed for the rapid detection of dengue infections. Dengue rapid diagnostic test (anti-dengue IgM/ IgG RDT) kits yield results in less than 15 min, are simple to perform and, therefore, are used widely as a point-of-care test in outpatient clinics and emergency departments [11]. These kits are designed to detect the presence of anti-dengue IgM or IgG antibodies in the blood of patients with dengue. The IgM RDT kit can detect DENV-IgM as early as 3–5 days, and as late as 2–3 months, of illness in the case of a primary dengue infection, whereas the IgG RDT kit can detect disease after the 10th day. For secondary infections, all the IgM and IgG RDT kits can detect DENV within 1 to 2 days after the onset of symptoms. One of the limitations of the IgM/IgG RDT kit is its inability to detect DENV antibodies in the acute phase of DENV infection, since it takes 3–5 and 10–14 days for anti-DENV IgM and IgG antibodies, respectively, to become detectable. To overcome these limitations, a combination IgM/IgG and NS1 RDT kit was developed, which can detect DENV from day 1 up to day 9 after the onset of fever in samples from patients with primary or secondary dengue infections [12]. Nonstructural 1 protein (NS1) is a highly conserved glycoprotein present at high concentrations in the serum of patients with dengue during the early clinical phase of the disease [13].

Here, we selected commercially available Humasis, SD Bioline, and CareUS dengue NS1 and IgM/IgG RDTs. The performances of these three RDT kits were compared and evaluated using samples from children with acute dengue in Myanmar. Moreover, we evaluated the performances of these RDTs in patients with primary and secondary serological profiles and those classified by DENV diagnosis using WHO guidelines and DENV serotypes. The evaluations were performed using existing qRT-PCR- and IgM/IgG ELISA-based diagnostic tests as references.

## 2. Materials and methods

### 2.1 Subjects

**Fig 1:**
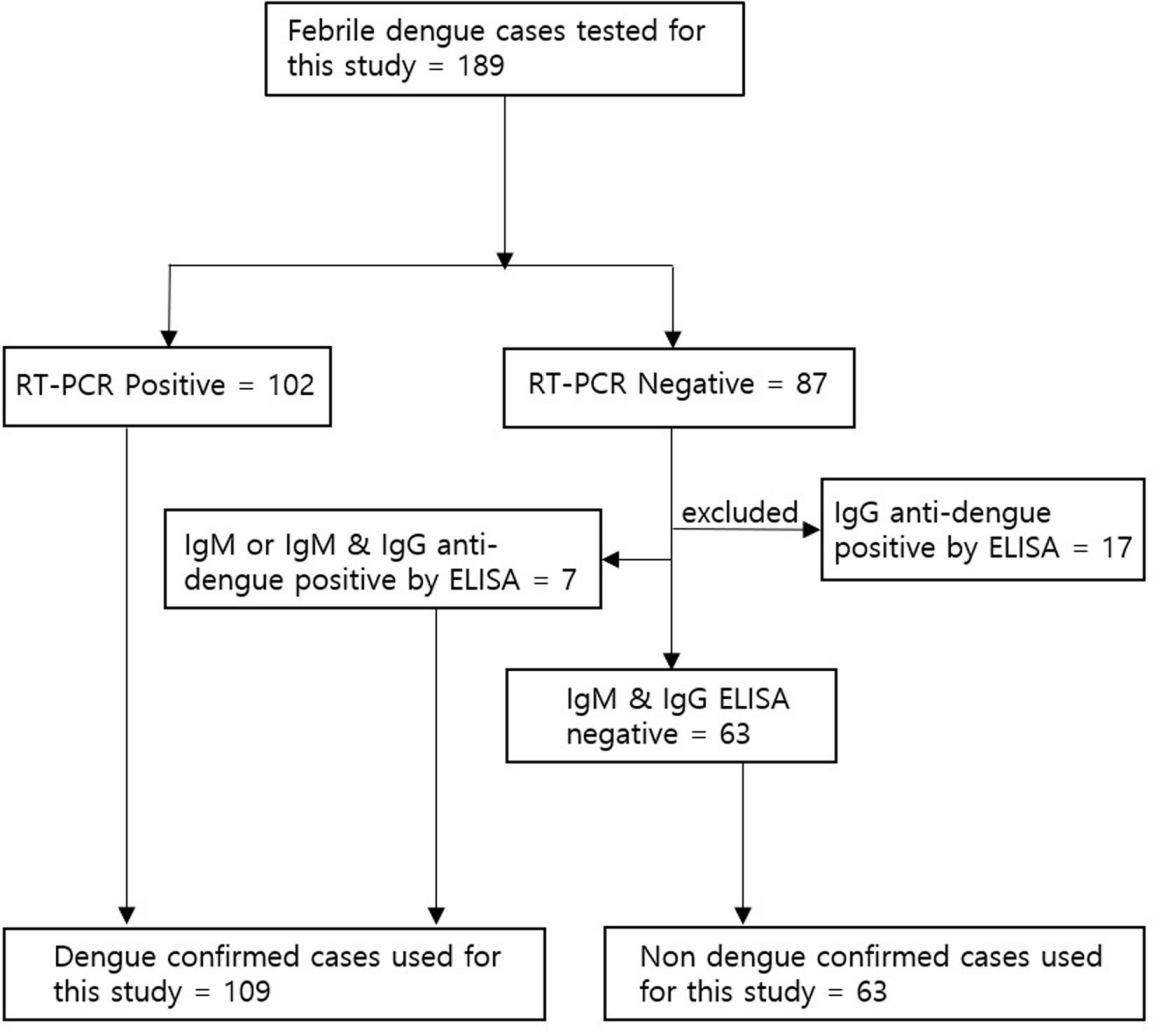
Flow diagram showing dengue-positive and *-*negative cases for the evaluation of the performances of three dengue rapid diagnostic test kits.

Blood samples were collected from patients with suspected dengue fever at the Yankin Children Hospital in Yangon in Myanmar from October 2015 to August 2016. Clinical diagnoses of dengue were categorized as dengue, dengue with warning signs, or severe dengue, according to the WHO guidelines [14]. A total of 189 samples were collected, of which 172 were included in this study (Fig 1). The serum panel consisted of 172 serum samples, including 102 cases confirmed to be positive for DENV infection by qRT-PCR and 7 cases confirmed to be positive for DENV infection based on IgM or IgM/IgG ELISAs. Additionally, the panel included 63 serum samples collected from patients with other illnesses that tested negative for DENV by qRT-PCR, or who tested negative for dengue by IgM and IgG ELISAs. The blood samples were collected on the first day of hospitalization. Serum samples were sent to the Laboratory of Diagnostic Tests of Korea University Guro Hospital for testing with dengue RDT and stored at -80°C until testing. This study was approved by the Medical Ethics Committee of Myanmar's Ministry of Health, the Department of Medical Research (64/Ethics 2015) and the Medical Ethics Committee of Korea University Guro Hospital (2017GR1767).

This protocol was designed based on in-house real-time RT-PCR and IgM/IgG ELISA. Dengue-positive and -negative cases were used for the comparative evaluation of the performances of three rapid diagnostic (RDT) kits: Humasis Dengue Combo NS1 and IgM/IgG (Humasis, Anyang, Korea), SD Bioline Dengue Duo NS1 and IgM/IgG (Standard Diagnostic, Yongin, Korea), and CareUS Dengue Combo NS1 & IgM/IgG (Wellsbio, Seoul, Korea).

### 2.2 qRT-PCR

The presence of dengue virus and the corresponding serotype was confirmed by qRT-PCR and sequencing. The theoretical specificity of the system was investigated using BLAST against the NCBI nucleotide database. RNA was extracted from the serum sample obtained from each patient with clinically diagnosed with dengue fever using the QIAamp Viral RNA kit (Qiagen, Germany) and used as template for qRT-PCR using a DiaStar OneStep Multiplex qRT-PCR Kit (Solgent, Korea). Newly designed DENV 1–4 primers and probes (S1 Table) were used in this study. The detection limit of the DENV 1–4 primers in qRT-PCR is shown in S2 Table. Amplification and detection were performed using a CFX-96 instrument (BioRad, USA). Thermocycling parameters were as follows: reverse transcription at 50°C for 30 min, inactivation at 95°C for 15 min, 40 cycles of fluorescence detection at 95°C for 40 s, and a final annealing at 62.7°C for 1 min. Samples were considered dengue-positive if target amplification occurred within 40 cycles. All PCR products were sequenced for DENV serotype confirmation.

### 2.3 Dengue IgM/IgG ELISA

The blood and serum samples were tested for dengue IgM/IgG using a Dengue IgG and IgM ELISA (MyBioSource Inc, San Diego, CA, USA) according to the manufacturer's instructions. Briefly, dengue serum samples were diluted 1:20 (IgG) and 1:1000 (IgM) in dilution buffer, and then 100 μL of the mixture was added to each well. After incubating for 10 min, the wells were washed and 100 μL of enzyme conjugate (anti-dengue IgM or IgG antibody labeled with peroxidase) was added to each well. After incubating for 10 min, the plates were washed and the conjugated complex was visualized by adding 100 μL of TMB solution to the wells. After incubating for 10 min, the reaction was stopped by adding 100 μL of stop solution, and the plate was then read at 450 nm using a microtiter plate reader. Positive determinations were made against the provided IgM and IgG reference sera (cutoff calibrators).

### 2.4 Humasis Dengue Combo NS1 and IgM/IgG

The Dengue Combo NS1 Ag and IgG/IgM Ab Rapid Test is a qualitative membrane-based immunoassay for the detection of NS1 antigen and IgG/IgM antibodies in human serum/plasma/whole blood. The test kit consists of one device for detecting the dengue NS1 antigen, and a second device for the differential detection of dengue IgG/IgM antibodies. All tests were performed in accordance with the manufacturer’s instructions. To detect the dengue NS1 antigen, 100 μL of whole blood, or serum/plasma sample, was added to the sample well of the Humasis Dengue NS1 device. Similarly, for the detection of dengue IgG/IgM, 10 μL of whole blood, or serum/plasma sample, was added to the sample well, followed by the addition of 3 to 4 drops (100–120 μL) of assay buffer to the round assay buffer well. All data were obtained after 15–20 min. Each cassette included a control and a test line, where the appearance of both the control and the test line indicated a positive result, and the appearance of the control line alone indicated a negative result.

### 2.5 SD Bioline Dengue Duo NS1 Ag and IgM/IgG test

The SD Bioline Dengue Duo NS1 Ag and IgG/IgM test kit is a one-step immunochromatographic assay designed to detect both dengue virus NS1 antigen and differential IgM/IgG antibodies to detect the presence of dengue virus in human whole blood, serum, and plasma. The SD Bioline Dengue Duo rapid test contains two tests in one device, where the left and right sides are dengue NS1 antigen and IgG/IgM tests, respectively. All tests in this study were carried out in accordance with the manufacturer’s instructions. To detect the dengue NS1 antigen, 100 μL of the test sample was added to the left sample well and the results were read at 15–20 min. To detect dengue IgG/IgM, 10 μL of test sample was added to the right sample well, and 4 drops (90–120 μL) of assay diluent were added to the round assay diluent well, and the results were read 15–20 min later. Each test included a control and a test line, where the appearance of both the control and test line indicated a positive result, while the appearance of only the control line indicated a negative result.

### 2.6 CareUS Dengue Combo NS1 and IgM/IgG

CareUS Dengue Combo NS1 and IgM/IgG tests (WellsBio, Seoul, Korea) are designed to diagnose dengue fever in acute to convalescent patients and consist of a dengue NS1 antigen test strip and an anti-dengue immune IgM/IgG test strip in one device. All tests were carried out in accordance with the manufacturer’s instructions. To detect the dengue NS1 antigen, 80 μL of the test sample was added to the left sample well, 1 drop (~40 μL) of the analytical diluent was then added to the same well, and the results were read at 20 min. To detect dengue IgG/IgM, 20 μL of test sample was added to the right sample well, followed by the addition of 1 drop (40 μL) of analytical diluent to the same well, and the results were read at 15–20 min. Each test included control and test lines, where the appearance of both the control and test line indicated a positive result, and the appearance of only the control line indicated a negative result.

### 2.7 Data analysis

Data were tabulated and analyzed using Microsoft Excel (Microsoft Inc., Redmond, WA, USA). Confidence intervals for sensitivity and specificity were set at 95% and a two-tailed p < 0.05 indicated significance. Statistical analysis was performed with Stata v13 (StataCorp, Texas, USA). The sensitivity and specificity for the assays were calculated based on “true positive dengue samples” (PCR and IgM & IgG positives) using a diagnostic test evaluation calculator program (https://www.medcalc.org/calc/diagnostic_test.php) and the Free Statistics Calculators program (https://www.danielsoper.com/statcalc/calculator.aspx?id=29).

## 3. Results

A total of 220 samples were assessed in this study. For the dengue-positive cases, there were 109 samples from children with acute symptomatic dengue confirmed by RT-PCR, IgM, and IgM/IgG ELISA (Fig 1). The patient included an almost equal ratio of males (52.29%) and females (47.71%). The mean age of the patients was 7.5 years (range 1–14 years). Acute phase samples from patients with suspected dengue were collected after 3 to 7 days of fever. To distinguish between primary and secondary dengue virus infections, we used a cutoff IgM/IgG ratio of 1.2, which was consistent with the published cutoff ratio range [15, 16]. There were 22 primary (20.2%) and 87 secondary infections (79.8%) among the dengue-positive cases. The serotype distribution of the 102 RT-PCR-based positive samples were DENV1 (77; 75.5%), DENV2 (23; 22.5%), and DENV4 (2; 2%). According to WHO guidelines, dengue-positive samples were classified as dengue (19; 17.4%), warning signs (63; 57.7%), or severe dengue (27; 24.7%). The dengue-negative cases yielded 63 samples from children with febrile non-dengue infections. All these samples were negative for dengue based on qRT-PCR and an anti-dengue IgM and IgG ELISA (Fig 1).

The left, middle, and right panels show NS1, IgM, and IgG RDT tests, respectively. The numbers in parenthesis below the x-axes indicate the number of patients with dengue according to the dengue classifications based on WHO guidelines.

### 3.1 Clinical performances of three RDT kits for acute dengue infection

The sensitivity and specificity for the NS1 antigen, IgM, and IgG for the three RDTs tested (Humasis, SD Bioline and CareUS) are shown in Table 1. Based on RT-PCR, CareUS Dengue Combo NS1 and IgM/IgG was the most sensitive (79.82%) for NS1 antigen detection followed by the Humasis Dengue Combo NS1 and IgM/IgG test (63.30%), and then the SD Bioline Dengue Duo NS1 Ag and IgM/IgG test (48.62%). The specificity of all three RDT kits for the NS1 antigen was 100%.

**Table 1:** Sensitivity and specificity of three commercial NS1 and IgM/IgG rapid diagnostic test kits.

Company	Test	% Sensitivity [95% CI]	% Specificity [95% CI]
**Humasis**	NS1	63.30 (69/109) [53.53–72.33]	100 (63/63) [94.31–100.00]
	IgM	51.38 (56/109) [41.61–61.06]	98.21 (62/63) [91.47–99.96]
	NS1 or IgM	81.65 (89/109) [73.09–88.42]	98.21 (62/63) [91.47–99.96]
	IgG	72.48 (79/109) [63.10–80.60]	95.24 (60/63) [86.71–99.01]
**SD Bioline**	NS1	48.62 (53/109) [38.94–58.39]	100 (63/63) [94.31–100.00]
	IgM	60.55 (66/109) [50.73–69.78]	100 (63/63) [94.31–100.00]
	NS1 or IgM	80.73 (88/109) [72.07–87.66]	100 (63/63) [94.31–100.00]
	IgG	77.98 (85/109) [69.03–85.35]	100 (63/63) [94.31–100.00]
**CareUS**	NS1	79.82 (87/109) [71.05–86.90]	100 (63/63) [94.31–100.00]
	IgM	89.91 (98/109) [82.66–94.85]	100 (63/63) [94.31–100.00]
	NS1 or IgM	96.33 (105/109) [90.87–98.99]	100 (63/63) [94.31–100.00]
	IgG	82.57 (90/109) [74.13–89.17]	100 (63/63) [94.31–100.00]

Based on the Dengue IgG and IgM ELISA (MyBioSource Inc., San Diego, CA, USA) as a reference, the CareUS Dengue Combo NS1 and IgM/IgG test was found to have the highest sensitivity (89.91%) for IgM antibodies followed by the SD Bioline Dengue Duo NS1 Ag and IgM/IgG test (60.55%), and then the Humasis Dengue Combo NS1 and IgM/IgG test (51.38%). The specificity of the CareUS Dengue Combo NS1 and IgM/IgG and the SD Bioline Dengue Duo NS1 Ag and IgM/IgG tests were 100% for IgM, while the specificity of the Humasis kit for IgM was 98.21%.

Based on the Dengue IgG and IgM ELISA (MyBioSource Inc., San Diego, CA, USA) as a reference, the CareUS Dengue Combo NS1 and IgM/IgG test was found to have the highest sensitivity (82.57%) for IgG antibodies, followed by the SD Bioline Dengue Duo NS1 Ag and IgM/IgG test (77.98%), and then the Humasis Dengue Combo NS1 and IgM/IgG test (72.48%). The specificities of the CareUS Dengue Combo NS1 and IgM/IgG and the SD Bioline Dengue Duo NS1 Ag and IgM/IgG tests were 100% for IgG, while the specificity of the Humasis kit for IgG was 95.24%.

### 3.2 Clinical performance of the three dengue RDTs in patients with primary and secondary serological profiles

Since the concentrations of NS1 and DENV IgM/IgG antibodies in the blood depends on whether the infection is primary or secondary, the sensitivities of three DENV RDT kits were determined in patients with primary or secondary serological profiles (Table 2). For NS1-based tests, all three NS1 RDT kits were more sensitive for primary infections (Humasis, 77.27%; SD Bioline, 72.73%; CareUS, 90.91%) than secondary infections (Humasis, 59.77%; SD Bioline, 42.53%; CareUS, 77.01%). For IgM-based tests, Humasis and SD Bioline IgM RDT kits had a higher sensitivity for primary infections (Humasis, 68.18%; SD Bioline, 72.73%) than secondary infections (Humasis, 47.13%; SD Bioline, 57.47%), while the CareUS IgM RDT kit had similar high sensitivities for both primary (86.36%) and secondary infections (90.80%). However, there was no statistically significant difference between primary infections and secondary infections using the three IgM RDT kits (P = 0.0961, 0.2284, and 0.6912, respectively). When the sensitivity of the two biomarkers (NS1 or IgM) was analyzed according or primary or secondary infection classifications, the difference in sensitivities between primary and secondary infections was drastically reduced for the Humasis (86.36 and 80.46%, respectively) and SD Bioline kits (90.91 and 78.16%, respectively), whereas the CareUS kit had similar sensitivities (90.91 and 97.70%) for both primary and secondary infections. There were no statistically significantly differences in all the NS1/IgM tests (P = 0.7589, 0.2344, and 0.1807 for the Humasis, SD Bioline, and CareUS kits, respectively).

**Table 2:** Sensitivity of three dengue rapid diagnostic tests for patients with primary and secondary serological profiles.

Status	Total (n)		% Sensitivity [95% CI]		
			Humasis	SD Bioline	CareUS
**Primary dengue**	22	NS1	77.27 (17/22) [54.63–92.18]	72.73 (16/22) [49.78–89.27]	90.91 (20/22) [70.84–98.88]
		IgM	68.18 (15/22) [45.13–86.14]	72.73 (16/22) [49.78–89.27]	86.36 (19/22) [65.09–97.09]
		NS1 or IgM	86.36 (19/22) [65.09–97.09]	90.91 (20/22) [70.84–98.88]	90.91 (20/22) [70.84–98.88]
		IgG	31.82 (7/22) [13.86–54.87]	40.91 (9/22) [20.71–63.65]	31.82 (7/22) [13.86–54.87]
		NS1 or IgM + IgG^**-**^ (% correctly classified)	63.64 (14/22) [40.66–82.80]	50.00 (11/22) [28.22–71.78]	59.09 (13/22) [36.35–79.29]
**Secondary dengue**	87	NS1	59.77 (52/87) [48.71–70.15]	42.53 (37/87) [31.99–53.59]	77.01 (67/87) [66.75–85.36]
		IgM	47.13 (41/87) [36.33–58.13]	57.47 (50/87) [46.41–68.01]	90.80 (79/87) [82.68–95.95]
		NS1 or IgM	80.46 (70/87) [70.57–88.19]	78.16 (68/87) [68.02–86.31]	97.70 (85/87) [91.94–99.72]
		IgG	82.76 (72/87) [73.6–90.02]	87.36 (76/87) [78.50–93.52]	95.40 (83/87) [[88.64–98.73]
		NS1 or IgM + IgG^**+**^ (% correctly classified)	65.52 (57/87) [54.56–75.39]	68.97 (60/87) [58.14–78.45]	95.40 (83/87) [88.64–98.73]
**P value**^a^		NS1	0.1458	0.0161	0.2337
		IgM	0.0967	0.2284	0.6912
		NS1 or IgM	0.7589	0.2344	0.1807
		IgG	<0.0001	<0.0001	<0.0001
		NS1 or IgM + IgG^**±**^	1*	0.1323*	<0.0001*

^a^Fisher’s exact test

*P values between primary and secondary infection.

The sensitivities of all three IgG kits were statistically significantly (p <0.05) higher for secondary infections (Humasis, 82.76%; SD Bioline, 87.36%; CareUS, 95.40%) than for primary infections (Humasis, 31.82%; SD Bioline, 40.91%; CareUS, 31.82%). When we analyzed the sensitivity of all biomarkers according to the classification as a primary (NS1/IgM/IgG^-^) or a secondary infection (NS1/IgM/IgG^+^), the Humasis RDT kit had similar sensitivities for primary (63.64%) and secondary infections (65.52%). However, the sensitivities of the SD Bioline (68.97%) and CareUS (95.40%) RDT kits for secondary infections were higher than for primary infections (SD Bioline, 50.00%; CareUS, 59.09%). Among the three RDT kits, the CareUS RDT kit was the only kit displaying a statistically significant difference between primary and secondary DENV infections (p < 0.05).

### 3.3 Clinical performance of the three RDT kits according to the day after the onset of fever

**Fig 2:**
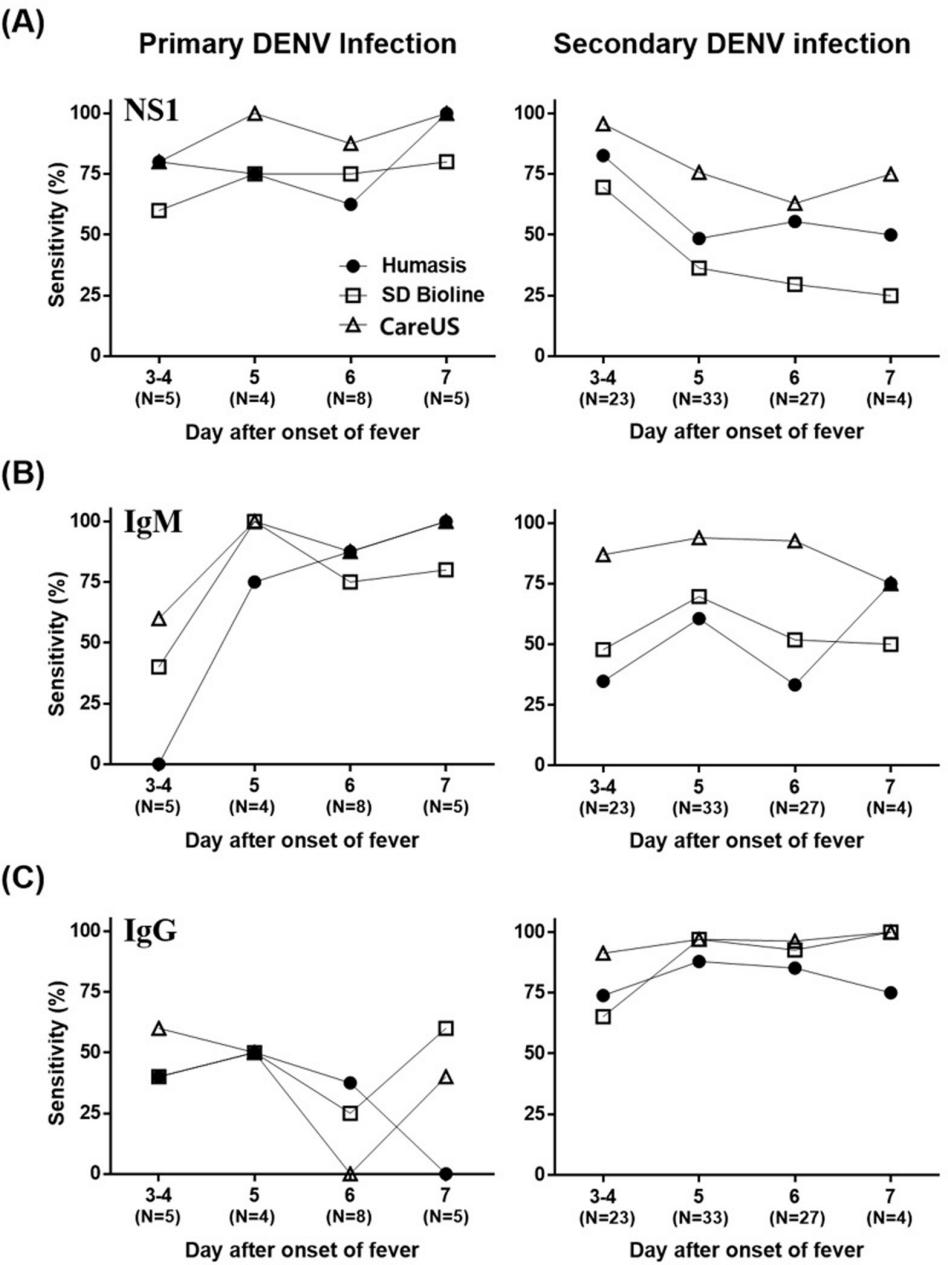
Comparison of the sensitivity of the three commercial kits against samples from patients with laboratory-confirmed dengue in relation to the day of onset of fever. **(A-C)** The sensitivity for (A) NS1, (B) IgM, and (C) IgG in primary (left panel) and secondary infections (right panel). The numbers in parenthesis below the x-axes indicate the number of patients with dengue assessed at each time point.

When comparing the sensitivities of the three NS1 RDT kits for primary and secondary infections according to day after onset of fever, all three NS1 RDT kits had continuously high sensitivities for primary infection during the entire febrile period, whereas the sensitivities of all three NS1 RDT kits for secondary infections gradually decreased with increasing days after the onset of fever (Fig 2A). The CareUS NS1 RDT kit had the highest sensitivity for both primary and secondary infections during the entire febrile period. The sensitivities of all three IgM RDT kits for primary infections drastically increased at the 5th day of fever, whereas the sensitivities of all three kits for secondary infections remained continuously high and did not change significantly during the entire febrile period (Fig 2B). For primary infections, the three IgG RDT kits had very low sensitivities during the congested pattern during the entire febrile period. In contrast, the sensitivities of all three IgG RDT kits for secondary infections remained continuously high during the entire febrile period (Fig 2C).

### 3.4 Real-time PCR threshold cycle (Ct) levels of dengue RDT kits for dengue-positive cases

For all the NS1 antigen tests, the Ct values for NS1 antigen-positive cases were statistically significantly lower (p < 0.05, except CareUS with a p = 0.0611) than for NS1 antigen-negative cases (Table 3). Among the three NS1 kits, the Ct values (32.18 ± 8.70) for the NS1 antigen-positive cases (N = 87) tested by the CareUS kit were higher than those of the other RDT kits. In the case of the IgM tests, there was no statistically significant difference (p < 0.05) between IgM-positive and IgM-negative cases for all the test kits, despite the Ct values of the IgM-positive cases being lower than those of the IgM-negative cases. Meanwhile, the Ct values of the IgG positive cases for all the IgG tests were statistically significantly (p < 0.05, except for SD Bioline with a p = 0.0632) higher than those of the IgG-negative cases.

**Table 3:** Performances of three rapid diagnostic tests in the detection of dengue NS1, IgM, and IgG compared to Ct values from RT-PCR.

Test	Company	Number of true positives	RDT-positive mean Ct [SD]	Number of false negatives	RDT-negative mean Ct [SD]	*P value
**NS1**	Humasis	69/102	31.06 [9.51]	33/102	36.24 [3.20]	0.0014
	SD Bioline	53/102	30.47 [9.82]	49/102	35.18 [5.60]	0.0027
	CareUS	87/102	32.18 [8.70]	15/102	35.94 [5.31]	0.0611
**IgM**	Humasis	52/102	31.94 [9.43]	50/102	33.56 [7.10]	0.3183
	SD Bioline	60/102	31.97 [9.44]	42/102	33.82 [6.53]	0.2690
	CareUS	93/102	32.42 [8.65]	9/102	35.91 [3.47]	0.2100
**IgG**	Humasis	74/102	34.33 [6.92]	28/102	28.51 [10.36]	0.0025
	SD Bioline	79/102	33.55 [7.44]	23/102	29.92 [10.71]	0.0632
	CareUS	85/102	33.60 [7.46]	17/102	28.88 [12.36]	0.0326

*P values between RDT-true positives and RDT- false negatives

### 3.5 Clinical performances of the three RDT kits according to WHO clinical classification

**Fig 3:**
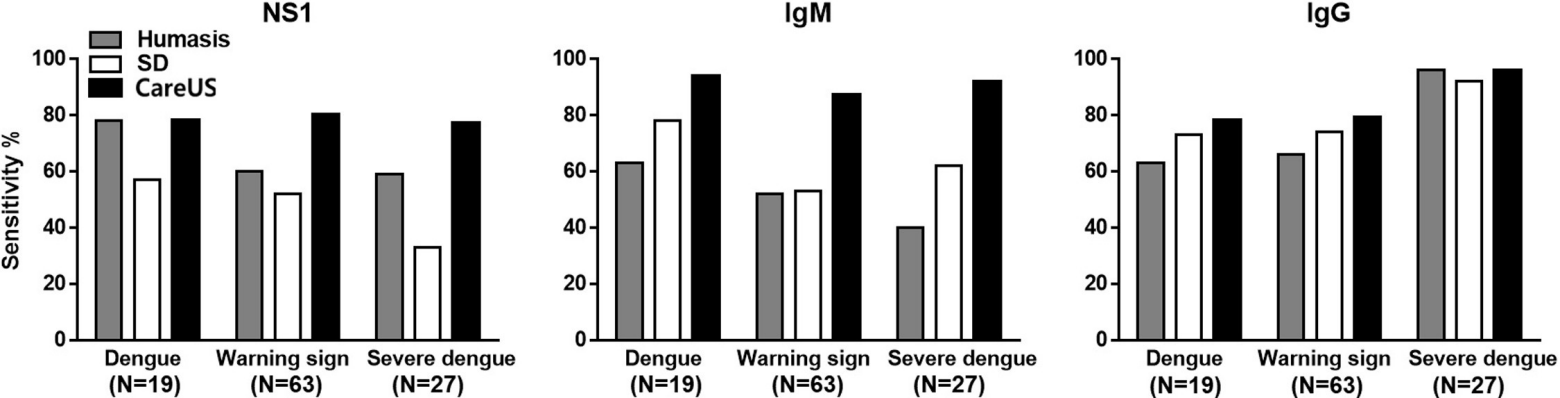
Sensitivity results from the three dengue RDT kits according to WHO guideline-based dengue classifications.

The clinical disease classifications, according to the WHO classifications, consisted of dengue (19; 17.43%), warning signs (63; 57.79%), and severe dengue (27; 24.77%), as shown in Fig 3. For the NS1 tests, the sensitivities of the Humasis and CareUS kits were 78.94% for cases classified as dengue, while the SD Bioline kit had a sensitivity as low as 57.89%. For the cases classified as warning signs and severe dengue, CareUS had the highest sensitivities of 80.95 and 77.77%, respectively, compared to Humasis (60.31 and 59.25%, respectively) and SD Bioline (52.38 and 33.33%, respectively). The Humasis NS1 and SD Bioline NS1 kits had higher sensitivities, which were in the order dengue, warning signs, and severe dengue. The CareUS NS1 kit had similarly high sensitivities for all three categories. With respect to the IgM test for the WHO classifications of dengue, warning signs, and severe dengue, the CareUS kit had the highest sensitivities of 94.73, 87.30, and 92.59% compared to the SD Bioline sensitivities of 78.94, 53.96, and 62.96%, and the Humasis sensitivities of 63.15, 52.38, and 40.74%, respectively. The Humasis IgM kit had the highest sensitivities, which were in the order dengue, warning signs, and severe dengue. The sensitivities of the SD Bioline IgM kit were in the order dengue, severe dengue, and warning signs. The CareUS IgM kit had similarly high sensitivities for all three categories. With respect to the IgG test, all three tested kits had similar sensitivities for each of the WHO classifications of dengue, warning signs, and severe dengue. Of note, all three kits had high sensitivities, where were in the order severe dengue, warning signs, and dengue.

### 3.6 Comparison of the sensitivities of the three RDT kits according to dengue serotype

**Fig 4:**
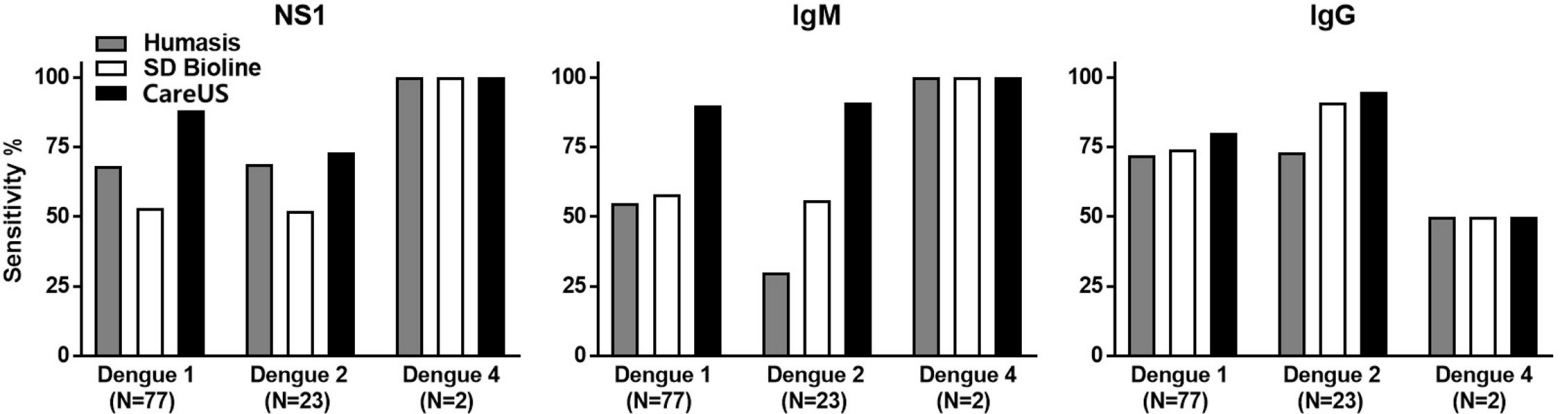
Comparison of the sensitivity of the three dengue NS1, IgM, and IgG RDT kits according to dengue virus serotypes. The left, middle, and right panels present NS1, IgM, and IgG RDT test results, respectively. The numbers in parenthesis below the x-axes indicate the number of patients with dengue according to dengue serotype.

The sensitivities of the three RDT kits for the dengue serotypes were compared and evaluated (Fig 4). For the NS1 test with respect to DENV1 and DENV2 cases, CareUS had the highest sensitivities (88.31 and 73.91%, respectively), followed by Humasis (68.83 and 69.56%, respectively), and SD Bioline (53.24 and 52.17%, respectively). For the IgM test with respect to DENV1 and DENV2 cases, CareUS had the highest sensitivities (90.90 and 91.30%, respectively), followed by the SD Bioline (58.44 and 56.52%, respectively) and Humasis kits (55.84 and 30.43%, respectively). The Humasis IgM kit had fairly low sensitivity to DENV2 than to DENV1, while the other two kits had similar sensitivities for DENV1 and DENV2. With respect to the two tests above, the IgG test from CareUS (80.51 and 95.65%) was more sensitive than SD Bioline (74.02 and 91.30%) and Humasis (72.72 and 73.91%) for DENV1 and DENV2, respectively. Unfortunately, we could not perform a comparative evaluation of the three RDT kits for DENV3, since there were no DENV3 samples in the dengue-positive group, and it was difficult to evaluate the sensitivities of the three RDT kits for DENV4 because of a lack of samples (N = 2).

## 4. Discussion

Dengue fever is caused by the dengue virus and is a tropical disease transmitted by mosquitoes [17, 18]. Secondary infections can lead to life-threatening dengue hemorrhagic fever or dengue shock syndrome. As stated by the WHO for the Southeast Asia and Western Pacific region, about 1.8 billion people are at risk of dengue, putting a significant economic and disease burden on these countries [19, 20]. Myanmar has seen an increase in dengue fever over the past decade, and is now classified as a high-risk dengue country in the Asia-Pacific region [21]. At present, a rapid diagnostic test kit for dengue fever can be used, but is in limited supply and rarely used in Myanmar [22]. In addition, information on circulating serotypes in Myanmar is limited because it requires PCR, which is only available in the National Health Laboratory under the Department of Public Health and the Department of Medical Research [8]. It was recently reported that DENV1 predominated in the 2013 outbreak, whereas serotypes 1, 2, and 4 were those mainly found in circulation in Myanmar in the 2015 outbreak [8]. In this study, we used DENV samples collected at the Yankin Children Hospital in Yangon in Myanmar from October 2015 to August 2016. DENV serotypes were identified in 102 DENV PCR-confirmed patients, where DENV1 (71.29%), DENV2 (21.29%), and DENV 4 serotypes (1.85%) were detected. DENV3 was not detected. In the 102 DENV PCR samples, DENV1 was found to be the most predominant. Our results are similar to the results reported in 2013, but not to results reported in 2015. The reason for this is probably because the results of the experiments reported by Oo et al. for 2015 [8] were derived from a small sample of 36 patients. In fact, the distribution of dengue type over the entire 2013–2015 period was similar to our results.

In this study, we compared and evaluated the diagnostic usefulness of three RDTs, Humasis, SD Bioline, and CareUS dengue kits based on dengue diagnosis criteria using RT-PCR and single serum for IgM/IgG. When the SD Bioline NS1 and IgM RDT kit results were compared with those from an earlier study [23], the sensitivity (80.73%) and specificity (100.00%) of the SD Duo NS1/IgM in this study were similar to those (88.65 and 98.75%) in recent studies [23]. The Humasis and CareUS DENV kits were the first tested in this study. Of the three DENV RDT kits in this study, CareUS had the highest sensitivity for NS1 (79.82%), IgM (89.91%), NS1/IgM (96.33%), and IgG (82.57%). Interestingly, the Humasis kit exhibited a higher sensitivity for NS1 than IgM, whereas the SD Bioline kit had a higher sensitivity for IgM than NS1. Combining the NS1 and IgM test results yielded the highest sensitivities for all RDT kits. The sensitivities of the SD Bioline and Humasis kits were markedly increased when the NS1 and IgM test results were combined.

It is important to distinguish between primary and secondary dengue fever when treating patients, because secondary infections can lead to dengue hemorrhagic fever and dengue shock syndrome with serious complications [24]. Primary and secondary infections have different NS1 antigen and IgM/IgG antibody profiles [25]. It has been shown in longitudinal studies on patients with dengue that patients remain NS1 antigen-positive after the DENV RNA amplicons disappear [26–28], probably because of the longer half-life of the NS1 protein. However, it has been reported that the NS1 detection test had low sensitivity for secondary dengue infections due to the immune complex formation of the NS1 antigen with pre-existing antibodies [29]. Indeed, the sensitivities of the three NS1 RDT kits in this study also gradually decreased for secondary dengue infections as the length of illness increased for all test kits, whereas the detection of NS1 in primary dengue remained high during the entire febrile period. However, the three NS1 RDT kits had high sensitivities for both primary and secondary DENV infections within 4 days of onset of fever. For the IgM tests, the sensitivities of all three IgM RDT kits for primary infections drastically increased after 5 days of illness, whereas the sensitivity for secondary infections remained high without significantly changing during the entire febrile period, suggesting that NS1 and DENV IgM detect DENV during different periods of the DENV primary infection. In contrast, the three IgG RDT kits had very low sensitivities for the congested pattern in primary DENV infections during the entire febrile period, whereas the sensitivities of all three IgG RDT kits for secondary infections remained high during the entire febrile period. In particular, when analyzing the sensitivities for all biomarkers according to primary (NS1/IgM/IgG^-^) and secondary infections (NS1/IgM/IgG^+^), the CareUS kit had statistically different (p <0.05) sensitivities of 59.09 and 95.40%, respectively. SD IgG kit showed no cross-reactivity with Chikungunya virus infected serum. All three NS1/IgM kits showed no cross reactivity with Chikungunya virus infected serum. However, in cross-reactivity test of three RDT kits against Chikungunya virus infected serum samples (n = 15) among other flavivirus, Humasis and CareUS IgG test showed cross-reactivities 80% and 73.3%, respectively (S2 Table). All three NS1/IgM kits showed no cross-reactivity with Chikungunya virus infected serum samples. Although we did not perform cross-reactivity testing of the three test kits for other flavivirus samples such as Japanese encephalitis virus (JEV), or Zika virus (ZIKV), which are common in southeast Asia, Humasis and CareUS IgG test might have cross-reactive activity with other flavivirus infected samples. Therefore, for confirmation of dengue infection only IgG is not recommended without additional NS1 Ag and IgM antibody status, and it should be checked whether another flavivirus is infected.

The threshold cycle (Ct) values reflect the overall amount of viral load in each clinical specimen, and the relative viral load can be estimated from the Ct value of each specimen. For specimens with low Ct values, the three RDT kits showed comparable sensitivities. However, for specimens with higher Ct values, the NS1 antigen and IgM of CareUS kit only detected Dengue viruses. These findings suggest that the CareUS kit detects lower viral loads more precisely than do the two other RDT kits.

The limitations of this study are that there were not enough DENV4 samples to analyze test performance and DENV3 samples were not collected. The results for adult samples may also be different, since only samples from children were used in this study. The sensitivity and specificity results in this study may be difference with the results classified by the standard dengue diagnosis (RT-PCR and paired serum for IgM/IgG) recommended by WHO, because dengue and non-dengue cases in this study were classified by using RT-PCR and single serum for IgM/IgG. In addition, the sensitivity of the three tested RDT kits may have decreased slightly because patient blood was stored at -80°C and shipped to Korea from Myanmar for testing.

In conclusion, our study suggests that testing for both dengue NS1 and IgM/IgG is more effective for diagnosis than either alone. RDTs are simple, easy to perform, inexpensive, sensitive, and require no sophisticated protocols, and, therefore can be used in any location. Among the kits evaluated, the CareUS RDT kit was the most efficient when confirming dengue infections by capturing the NS1 antigen and detecting DENV-IgG/IgM from infected patients. Moreover, it was the most convenient to use (as the results could be obtained in 15 min), easy to perform, and it did not require special laboratory equipment.

## Supporting information

S1 Table In-house primer and probe sequences used for dengue detection. (DOCX)

S2 Table The detection limit of DENV1-4 by qRT-PCR. (DOCX)

S3 Table Cross-reactivity test of the three RDT kits for Chikungunya virus. (DOCX)

S1 Row data (XLSX)

## References

[1] Murray NEA, Quam MB, Wilder-Smith A. Epidemiology of dengue: past, present and future prospects. Clinical epidemiology. 2013;5:299 10.2147/CLEP.S34440 23990732 PMC3753061

[2] Luo L, Liang Hy, Hu Ys, Liu Wj, Wang Yl, Jing Ql, et al Epidemiological, virological, and entomological characteristics of dengue from 1978 to 2009 in Guangzhou, China. Journal of Vector Ecology. 2012;37(1):230–40. 10.1111/j.1948-7134.2012.00221.x 22548558

[3] World Health Organization. Dengue and dengue haemorrhagic fever (http://www.who.int/mediacentre/factsheets/fs117/en/, accessed 15 March 2004).

[4] Calisher CH. Persistent emergence of dengue. Emerging infectious diseases. 2005;11(5):738 10.3201/eid1105.050195 15898171 PMC3320357

[5] Echaubard P, Rudge JW, Lefevre T. Evolutionary perspectives on human infectious diseases: Challenges, advances, and promises. Evolutionary applications. 2018;11(4):383–93. 10.1111/eva.12586 29636793 PMC5891049

[6] World Health Organization, Dengue and sever dengue (http://www.who.int/news-room/fact-sheets/detail/dengue-and-severe-dengue, accessed 13 September 2018).

[7] Rey JR. Dengue in Florida (USA). Insects. 2014;5(4):991–1000. 10.3390/insects5040991 26462955 PMC4592614

[8] Oo PM, Wai KT, Harries AD, Shewade HD, Oo T, Thi A, et al The burden of dengue, source reduction measures, and serotype patterns in Myanmar, 2011 to 2015–R2. Tropical medicine and health. 2017;45(1):35. 29118655 10.1186/s41182-017-0074-5 PMC5667489

[9] Teparrukkul P, Hantrakun V, Day NP, West TE, Limmathurotsakul D. Management and outcomes of severe dengue patients presenting with sepsis in a tropical country. PloS one. 2017;12(4):e0176233 10.1371/journal.pone.0176233 28437459 PMC5402971

[10] Shamala D. Laboratory Diagnosis of Dengue: A Review. International Medical Journal Malaysia. 2015;14(1).

[11] Jusoh M, Akmalina TN, Shueb RH. Performance Evaluation of Commercial Dengue Diagnostic Tests for Early Detection of Dengue in Clinical Samples. Journal of Tropical Medicine. 2017;2017. 10.1155/2017/4687182 PMC5742879 29379526

[12] Young PR, Hilditch PA, Bletchly C, Halloran W. An antigen capture enzyme-linked immunosorbent assay reveals high levels of the dengue virus protein NS1 in the sera of infected patients. Journal of clinical microbiology. 2000;38(3):1053–7. 10698995 10.1128/jcm.38.3.1053-1057.2000 PMC86336

[13] Alcon S, Talarmin A, Debruyne M, Falconar A, Deubel V, Flamand M. Enzyme-linked immunosorbent assay specific to Dengue virus type 1 nonstructural protein NS1 reveals circulation of the antigen in the blood during the acute phase of disease in patients experiencing primary or secondary infections. Journal of clinical microbiology. 2002;40(2):376–81. 10.1128/JCM.40.2.376-381.2002 11825945 PMC153354

[14] World Health Organization, Handbook for clinical management of dengue Geneva, Switzerland WHO 2012.

[15] de Souza VAUF, Tateno AF, Oliveira RR, Domingues RB, Araújo ES, Kuster GW, et al Sensitivity and specificity of three ELISA-based assays for discriminating primary from secondary acute dengue virus infection. Journal of clinical virology. 2007;39(3):230–3. 10.1016/j.jcv.2007.04.005 17509934

[16] Domingo C, de Ory F, Sanz JC, Reyes N, Gascón J, Wichmann O, et al Molecular and serologic markers of acute dengue infection in naive and flavivirus-vaccinated travelers. Diagnostic microbiology and infectious disease. 2009;65(1):42–8. 10.1016/j.diagmicrobio.2009.05.004 19679234

[17] Heilman JM, De Wolff J, Beards GM, Basden BJ. Dengue fever: a Wikipedia clinical review. Open medicine. 2014;8(4):e105 25426178 PMC4242787

[18] Chen LH, Wilson ME. Transmission of dengue virus without a mosquito vector: nosocomial mucocutaneous transmission and other routes of transmission. Clinical Infectious Diseases. 2004;39(6):e56–e60. 10.1086/423807 15472803

[19] Grover GS, Mahajan V, Thaware P, Takkar J. Dengue Death Review: A Tool to Adjudge the Cause of Dengue Mortality and Use of the Tool for Prevention of Dengue Deaths. World Academy of Science, Engineering and Technology, International Journal of Medical, Health, Biomedical, Bioengineering and Pharmaceutical Engineering. 2015;9(12):853–6.

[20] Mallhi TH, Khan AH, Sarriff A, Adnan AS, Khan YH. Determinants of mortality and prolonged hospital stay among dengue patients attending tertiary care hospital: a cross-sectional retrospective analysis. BMJ open. 2017;7(7):e016805 10.1136/bmjopen-2017-016805 28698348 PMC5724230

[21] Shepard DS, Undurraga EA, Halasa YA. Economic and disease burden of dengue in Southeast Asia. PLoS neglected tropical diseases. 2013;7(2):e2055 10.1371/journal.pntd.0002055 23437406 PMC3578748

[22] Thu HM, Lowry K, Myint TT, Shwe TN, Han AM, Khin KK, et al Myanmar dengue outbreak associated with displacement of serotypes 2, 3, and 4 by dengue 1. Emerging Infectious Diseases. 2004;10(4):593 10.3201/eid1004.030216 15200847 PMC3323074

[23] Wang SM, Sekaran SD. Early diagnosis of Dengue infection using a commercial Dengue Duo rapid test kit for the detection of NS1, IGM, and IGG. The American journal of tropical medicine and hygiene. 2010;83(3):690–5. 10.4269/ajtmh.2010.10-0117 20810840 PMC2929071

[24] Changal KH, Raina A, Raina M, Bashir R, Latief M, Mir T, et al Differentiating secondary from primary dengue using IgG to IgM ratio in early dengue: an observational hospital based clinico-serological study from North India. BMC infectious diseases. 2016;16(1):715 10.1186/s12879-016-2053-6 27894268 PMC5127094

[25] Vickers IE, Harvey KM, Brown MG, Nelson K, DuCasse MB, Lindo JF. The performance of the SD BIOLINE Dengue DUO® rapid immunochromatographic test kit for the detection of NS1 antigen, IgM and IgG antibodies during a dengue type 1 epidemic in Jamaica. Journal of biomedical science. 2015;22(1):55. 26173484 10.1186/s12929-015-0164-9 PMC4502463

[26] Pok K-Y, Lai Y-L, Sng J, Ng L-C. Evaluation of nonstructural 1 antigen assays for the diagnosis and surveillance of dengue in Singapore. Vector-Borne and Zoonotic Diseases. 2010;10(10):1009–16. 10.1089/vbz.2008.0176 20426686 PMC2992696

[27] Bessoff K, Phoutrides E, Delorey M, Acosta LN, Hunsperger E. Utility of a commercial nonstructural protein 1 antigen capture kit as a dengue virus diagnostic tool. Clinical and vaccine immunology. 2010;17(6):949–53. 10.1128/CVI.00041-10 20410325 PMC2884419

[28] Dussart P, Petit L, Labeau B, Bremand L, Leduc A, Moua D, et al Evaluation of two new commercial tests for the diagnosis of acute dengue virus infection using NS1 antigen detection in human serum. PLoS neglected tropical diseases. 2008;2(8):e280 10.1371/journal.pntd.0000280 18714359 PMC2500180

[29] Koraka P, Burghoorn-Maas CP, Falconar A, Setiati TE, Djamiatun K, Groen J, et al Detection of immune-complex-dissociated nonstructural-1 antigen in patients with acute dengue virus infections. Journal of clinical microbiology. 2003;41(9):4154–9. 10.1128/JCM.41.9.4154-4159.2003 12958240 PMC193852

