# Supplementary material for: Comparative evaluation of three dengue duo rapid test kits to detect NS1, IgM, and IgG associated with acute dengue in children in Myanmar
Source: PLoS One. 2019 Mar 13;14(3):e0213451. doi: 10.1371/journal.pone.0213451 (PMC6415848; doi:10.1371/journal.pone.0213451)
Supplement: S1 Table — (DOCX) [file pone.0213451.s001.docx]

S1 Table. In-house primer and probe sequences used for dengue detection

| Primer name | Primer sequence (5′ → 3′) | Position |
| --- | --- | --- |
| DENV 1 forward | AGTGCCATHGCYCAAGCTA | 5095 |
| DENV 1 reverse | GGCTCCCACAAGRGTTGT | 5274 |
| DENV 1 probe | [FAM]CATAGTCCGTGAGGCCATAAAAAG[BHQ1] | 5140 |
| DENV 2 forward | AGTTGTGTGACGACGATGGC | 85 |
| DENV 2 reverse | GTTGCCCAACACAAGGGGAA | 218 |
| DENV 2 probe | [HEX]CAAACAACCTGCCACTCTAAGGAA[BHQ1] | 150 |
| DENV 3 forward | TCAGAAACGCAGCATGGGAC | 1771 |
| DENV 3 reverse | GATCACAGCCAACCCAGTGG | 1887 |
| DENV 3 probe | [CY5]AGATGCACCTTGCAAGATTCCTTTCT[BHQ1] | 1821 |
| DENV 4 forward | AGAAGAGCTGGAGAAACTG | 8181 |
| DENV 4 reverse | GATGYTGTTGAACAGGTTCAC | 8321 |
| DENV 4 probe | [TexasRed]GCGTCGGGAAACATYGTGAGYG[BHQ1] | 8280 |
