# Supplementary material for: Comparative evaluation of three dengue duo rapid test kits to detect NS1, IgM, and IgG associated with acute dengue in children in Myanmar
Source: PLoS One. 2019 Mar 13;14(3):e0213451. doi: 10.1371/journal.pone.0213451 (PMC6415848; doi:10.1371/journal.pone.0213451)
Supplement: S2 Table — (DOCX) [file pone.0213451.s002.docx]

S2 Table. The detection limit of DENV1-4 by qRT-PCR

| Plasmid Copy Number (copies/reaction) | Ct value | | | |
| --- | --- | --- | --- | --- |
|  | DENV1 | DENV2 | DENV3 | DENV4 |
| 10⁸ | 12.17 | 13.21 | 16.34 | 11.44 |
| 10⁷ | 17.16 | 17.39 | 19.42 | 14.94 |
| 10⁶ | 20.09 | 21.24 | 22.86 | 19.53 |
| 10⁵ | 23.75 | 25.03 | 25.83 | 22.93 |
| 10⁴ | 26.90 | 28.65 | 27.48 | 25.49 |
| 10³ | 29.29 | 32.01 | 31.01 | 29.56 |
| 10² | 32.71 | 35.28 | 34.20 | 32.61 |
| 10¹ | 36.16 | 36.66 | 36.18 | 35.47 |
| 10^0^ | 39.42 | N/A | N/A | 39.04 |
