# Supplementary material for: Comparative evaluation of three dengue duo rapid test kits to detect NS1, IgM, and IgG associated with acute dengue in children in Myanmar
Source: PLoS One. 2019 Mar 13;14(3):e0213451. doi: 10.1371/journal.pone.0213451 (PMC6415848; doi:10.1371/journal.pone.0213451)
Supplement: S3 Table — (DOCX) [file pone.0213451.s003.docx]

S3 Table. Cross-reactivity test of the three RDT kits for Chikungunya virus

| Samples | Total (n) |  | Cross-reactivity % | | |
| --- | --- | --- | --- | --- | --- |
|  |  |  | Humasis | SD Bioline | CareUS™ |
| Serum samples from patients infected with Chikungunya virus* | 15 | NS1 | 0 (0/15) | 0 (0/15) | 0 (0/15) |
|  |  | IgM | 0 (0/15) | 0 (0/15) | 0 (0/15) |
|  |  | IgG | 80 (12/15) | 0 (0/15) | - 1. (11/15) |

*Serum samples purchased from TRINA BIOREACTIVES AG (Swiss)
